# Cost-related medication nonadherence among over-indebted individuals enrolled in statutory health insurance in Germany: a cross-sectional population study

**DOI:** 10.1186/s12913-019-4710-0

**Published:** 2019-11-26

**Authors:** Jacqueline Warth, Marie-Therese Puth, Judith Tillmann, Niklas Beckmann, Johannes Porz, Ulrike Zier, Klaus Weckbecker, Birgitta Weltermann, Eva Münster

**Affiliations:** 1 Institute of General Practice and Family Medicine, University Hospital Bonn, Venusberg-Campus 1, 53127 Bonn, Germany; 2 Department of Medical Biometry, Informatics and Epidemiology (IMBIE), University Hospital of Bonn, Venusberg-Campus 1, 53127 Bonn, Germany; 3 Faculty of Medicine, Institute of General Practice, University of Düsseldorf, Düsseldorf University Hospital, Postfach 10 10 07, 40001 Düsseldorf, Germany

**Keywords:** Nonadherence, Over-indebtedness, Debt, Socioeconomic status, Delinquency, Health inequality, Access to health care, Prescription, Medication

## Abstract

**Background:**

Millions of citizens in high-income countries face over-indebtedness that implies being unable to cover payment obligations with available income and assets on an ongoing basis. Studies have shown an association between over-indebtedness and health outcomes, independent of standard socioeconomic status measures. Patterns of cost-related medication nonadherence (CRN) among over-indebted individuals are yet unclear. The aim of this study was to examine the frequency of nonadherence to prescribed medications due to cost, and to identify risk factors for CRN among over-indebted individuals in Germany.

**Methods:**

In 2017, we conducted a cross-sectional survey among over-indebted individuals recruited in 70 debt advice agencies in North Rhine-Westphalia, Germany. Data on CRN in the last 12 months (i.e. not filling prescriptions, skipping or decreasing doses of prescribed medication due to financial problems) were collected by a survey using a self-administered written questionnaire that was returned by 699 individuals with a response rate of 50.2%. Prevalence of CRN was assessed using descriptive statistics. Multiple logistic regression analysis was performed to examine risk factors of CRN, including participants enrolled in statutory health insurance with complete data (*n* = 521).

**Results:**

The prevalence of CRN was 33.6%. The chronically ill had significantly greater odds of cost-related medication nonadherence (aOR 1.96; 95% CI 1.27–3.03) than individuals without a chronic illness. CRN was more likely to occur in individuals who had discussed financial problems with their general practitioner (aOR 1.58; 95% CI 1.01–2.47). There was no association between CRN and other sociodemographic factors or socioeconomic status.

**Conclusions:**

Medication nonadherence due to financial pressures is common among over-indebted citizens enrolled in statutory health insurance in Germany. Stakeholders in social policy, research and health care need to address over-indebtedness to develop strategies to safeguard access to relevant medications, especially among those with high morbidity.

**Trial registration:**

Arzneimittelkonsum, insbesondere Selbstmedikation bei überschuldeten Bürgerinnen und Bürgern in Nordrhein-Westfalen (ArSemü), (engl. ‘Medication use, particularly self-medication among over-indebted citizens in North Rhine-Westphalia’), German Clinical Trials Register: DRKS00013100. Date of registration: 23.10.2017. Date of enrolment of the first participant: 18.07.2017, retrospectively registered.

## Background

Across European countries, about 10% of households are considered over-indebted, and this number continues to increase [1, 2]. In Germany alone, 6.9 million citizens are over-indebted which is defined as being unable to cover payment obligations and living expenses with available income and assets on an ongoing basis [3]. Over-indebtedness may reflect both a cause and consequence of poor health for individuals at all socioeconomic positions [4, 5]. Previous research has revealed an association between over-indebtedness and various physical and mental health outcomes [6–9] that was not explained by standard socioeconomic status (SES) measures such as income and education. Recently, a first register-based study among 48,778 Finnish adults examined the incidence of chronic disease using 15-year follow-up data on long-term over-indebted individuals and matched controls [10]: Over-indebtedness was associated with an increased risk of chronic diseases such as diabetes, bronchial asthma, coronary heart disease and psychoses. In line with these findings, the demand for medical care among the over-indebted is likely to be high.

Previous studies indicate that many patients restrict their medication use due to cost although most high-income countries provide various forms of coverage and co-payments for prescription drugs [11, 12]. Studies consistently found those with a low income and poor health, and individuals without drug coverage, to have an increased risk of cost-related medication nonadherence (CRN) [11–14]. However, evidence on how medication cost pressures affect the over-indebted is scarce, yet might be relevant to advance the understanding of inequalities in health and facilitate access to medical care [15].

Several studies suggest an association between measures of debt and cost-related medication nonadherence [16–18]. However, in contrast to over-indebtedness, the mere presence of debt does not reflect financial difficulties. Over-indebted individuals may be bound to weigh competing financial commitments and spending on necessities, such as essential medications when co-payments are required. In a longitudinal survey representative of US adults older than 50 years, over-indebted individuals were more likely to experience CRN over the 2-year follow-up [18]. In a cross-sectional study of 666 adults seeking debt counselling in Germany in 2010, a majority of over-indebted individuals restricted seeking medical care and filling prescriptions in the face of their financial strain [19]. Hence, the over-indebted may reflect a vulnerable population group that is especially susceptible to co-payments but has been largely neglected in health policy and health services research. In view of the fact that access to essential medicines is considered a component of the human right to health, and goal of national health systems, CRN reflects a critical public health issue [20]. Serious adverse health effects and increased use of medical services can arise from cost-related medication nonadherence [21–23].

In Germany, adults enrolled in statutory health insurance (SHI) are required to pay co-payments for medical services including medications. More specifically, co-payments for medications range from 5 to 10 euros per prescription, and any costs of over-the-counter drugs that are excluded from reimbursement. Patients can apply for reimbursement or waiver of co-payments if these exceed a 2 % ceiling of gross household income per annum. When patients’ attending physician attests a chronic condition, the ceiling can be reduced to 1 % (§ 62 German Social Code Book V). The objective of the present study is to examine the prevalence and factors associated with cost-related medication nonadherence among over-indebted individuals who are enrolled in statutory health insurance in Germany.

## Methods

This cross-sectional study among clients of debt advice agencies was conducted to examine medication use and self-medication in the over-indebted (OID survey; German acronym: ArSemü). In Germany, approved debt advice agencies provide debt and insolvency counselling services to over-indebted consumers (Insolvency Statute; German: Insolvenzordnung; §305). Between July and October 2017, 70 of 145 debt advice agencies throughout the federal state of North Rhine-Westphalia that all have a mandated role in insolvency proceedings and target over-indebted clients, served as recruiting offices. These agencies were associated with the local German Consumer Organisation or one of the member organisations of the ‘Expert Committee Debt Counselling of Non-statutory Welfare NRW’ (German: Fachausschuss Schuldnerberatung der Freien Wohlfahrtspflege NRW) that invited the agencies to conduct recruitment. The demographic structure of North Rhine-Westphalia, which is the most populous of the 16 federal states in Germany, is similar to the national average [23]. Reasons for non-participation of agencies were primarily lack of resources (time and staff) for recruitment.

**Fig. 1:**
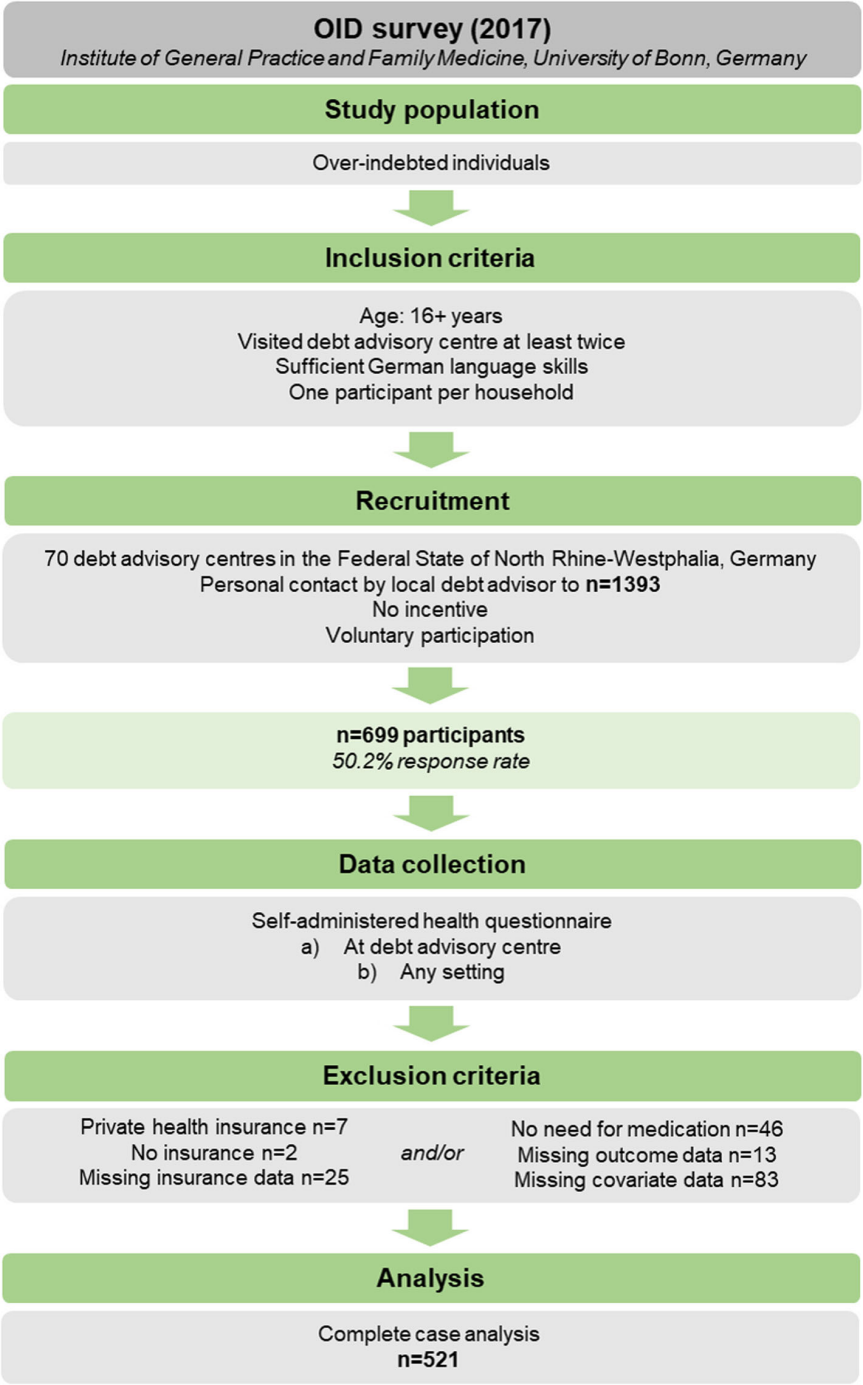
Flow chart of participants included in logistic regression analysis

A comprehensive study guide informed the debt advisors about study objectives, procedures and eligibility criteria in order to standardise recruitment. Eligibility criteria for over-indebted clients were a) having completed at least an initial consultation based on the premise that it reflects a sensitive situation necessary to build trust, b) at least 16 years of age to assure contractual capability, c) sufficient language, reading and writing skills to complete the questionnaire. Moreover, all nationalities but only one respondent within each household were considered eligible. When debt advisors identified clients as eligible, they invited clients to partcipate in the anonymous survey during the consultation in chronological order, and handed out the study material (standardised questionnaire, postage paid return envelope) (Fig. 1). Since approved debt advisory centres throughout Germany offer counselling specifically for over-indebted private households, all eligible clients were considered “over-indebted”.

We specifically designed a questionnaire for the survey among the over-indebted which was reviewed by selected debt advisors. To ensure validity and reliability of the questionnaire, we used several items from questionnaires that were developed for representative surveys of the adult population in Germany by the Robert Koch Institute, the national public health agency [24, 25], and surveys that examined access to medication in the German population [26], and in a population of over-indebted clients [19]. Questionnaires returned within one month after the end of recruitment were included in the analysis.

The questionnaire assessed cost-related underuse of medication and dietary supplements in the last 12 months. First, participants self-reported difficulty in obtaining medical products due to their financial situation (yes; no; no need for medication and dietary supplements). Second, the survey assessed specific CRN behaviours. Participants self-reported whether they had been delaying or not filling a prescription, skipping or decreasing doses of prescribed medications and skipping prescribed or over-the-counter medication for financial reasons in the previous year. In line with previous studies that have assessed test-retest reliability [27] and construct validity [28] for similar measures of CRN, a dichotomous outcome variable was generated to indicate subjects’ nonadherence to prescribed medications due to cost pressures. CRN was assumed when subjects reported not filling a prescription and/or skipping or decreasing doses of a prescribed medication.

According to a conceptual framework developed by Piette et al. [29], patients’ medication use and adherence in response to financial pressures is not only modified by patient characteristics and medication but also provider and health system factors. Therefore, analysis was limited to members of statutory health insurance. Due to a variety of tariffs, co-payments for privately insured individuals differ considerably from those enrolled in statutory health insurance in Germany. Hence, we excluded members of private health insurance (*n* = 7) a priori. Likewise, uninsured subjects (*n* = 2) and those with missing data on health insurance (*n* = 25) were excluded. Moreover, subjects who self-reported they “did not need medication or dietary supplements” in the last 12 months (*n* = 46) or missing outcome data (*n* = 13) were excluded from the analysis.

The role of providers in cost-related medication underuse was controlled for by taking into account whether patients had ever discussed financial problems with their general practitioner (yes, no). Moreover, sociodemographic characteristics, including age, sex, migration background, marital status, number of children, as well as socioeconomic status, and health status were considered as covariates in multiple logistic regression analysis. Age was classified into four age groups (18–29; 30–49; 50–64; 65–79 years) to differentiate phases of life. Immigrants and individuals who had at least one parent who was born outside of Germany were considered as having a migration background. We also controlled for marital status, classified into three groups, namely married, previously married (i.e. divorced or widowed), and never married subjects, and the number of children, categorised into four groups. Educational attainment was derived from questions about the highest general educational and vocational qualifications (low; medium; high) using the International Standard Classification of Education (ISCED) [30]. Current employment was assumed if subjects reported any kind of full, part-time or marginal employment. Those who did not report employment status but receiving unemployment benefits were considered unemployed. Moreover, individuals’ morbidity was controlled for in terms of self-reported chronic health conditions. Medical professionals examined any chronic health conditions respondents self-reported as free-text responses, and verified the data by comparing these to respondents’ self-reported health complaints underlying current medication use in the last 7 days. Based on the available data, medical professionals classified existing chronic conditions according to ICD-10-GM chapters (German adaptation of the International Statistical Classification of Diseases and Related Health Problems). The most common chronic conditions included endocrine, nutritional and metabolic diseases (Chapter IV; 20.9%), mental and behavioural disorders (Chapter V; 20.2%) and diseases of the circulatory system (Chapter IX; 19.5%). The presence or absence of a chronic health condition also gives some indication of subjects’ co-payment ceiling of one or 2 % of annual household income, respectively.

We used descriptive statistics to examine population characteristics and the prevalence of cost-related medication nonadherence among the over-indebted, and chi-squared test or Fisher’s exact test to examine differences in the distribution of characteristics across comparison groups. In order to examine factors that are associated with cost-related underuse of prescribed medication, crude and adjusted odds ratios (OR) and 95% confidence intervals (CI) were estimated using multiple logistic regression analysis. For logistic regression analysis, we performed a complete case analysis to handle missing data as the proportion of missing values in most variables was small and considered to be missing at random. In all models, the reference group for covariates was defined as the most frequent category, except for the reference category of age (youngest age group and chronic illness (absence of a chronic illness). All independent variables were entered into the model simultaneously. The level of statistical significance was set at 0.05. Analyses were carried out using IBM SPSS Statistics (version 25).

## Results

Debt advisors invited 1393 clients to participate in the OID survey. Of these, 699 individuals returned the questionnaire with complete data on sex and age which corresponds to a response rate of 50.2%. Following the exclusion of participants based on health insurance status, need for medical products and missing outcome data, the sample comprised 604 individuals. Table 1 presents characteristics of these participants, stratified by cost-related medication nonadherence. Participants’ mean age was 43.1 years (standard deviation 12.8 years; range 19–76 years). The prevalence of any cost-related medication nonadherence in the last 12 months among the over-indebted was 33.6% (*n* = 203). More specifically, the share of respondents that reported not filling a prescription was 26.8% (*n* = 162) while 13.4% (*n* = 81) reported skipping or decreasing doses of a prescribed medication due to cost.

**Table 1:** Characteristics of participants

			CRN^b^				
	Total sample (*n* = 604)^a^		Yes (n = 203)		No (*n* = 401)		Diff. between groups^c^
Variables	n	%	n	%	n	%	*p*-value
Age							0.173
18–29 years	107	17.7	41	20.2	66	16.5	
30–49 years	303	50.2	107	52.7	196	48.9	
50–64 years	156	25.8	47	23.2	109	27.2	
65–79 years	38	6.3	8	3.9	30	7.5	
Missing	0	0.0	0	0.0	0	0.0	
Gender							0.023
Female	348	57.6	130	64.0	218	54.4	
Male	256	42.4	73	36.0	183	45.6	
Missing	0	0.0	0	0.0	0	0.0	
Migrant background							0.923
No	343	56.8	116	57.1	227	56.6	
Yes	225	37.3	76	37.4	149	37.2	
Missing	36	6.0	11	5.4	25	6.2	
Marital status							0.119^d^
Married	131	21.7	34	16.7	97	24.2	
Previously married	232	38.4	83	40.9	149	37.2	
Never married	234	38.7	85	41.9	149	37.2	
Missing	7	1.2	1	0.5	6	1.5	
Number of children							0.668^d^
No children	176	29.1	58	28.6	118	29.4	
1 child	133	22.0	48	23.6	85	21.2	
2 children	154	25.5	54	26.6	100	24.9	
3 or more children	137	22.7	43	21.2	94	23.4	
Missing	4	0.7	0	0.0	4	1.0	
Education level							0.209^d^
Low	273	45.2	100	49.3	173	43.1	
Medium	304	50.3	95	46.8	209	52.1	
High	26	4.3	7	3.4	19	4.7	
Missing	1	0.2	1	0.5	0	0.0	
Employment status							0.964^d^
Employed	307	50.8	102	50.2	205	51.1	
Unemployed	287	47.5	98	48.3	189	47.1	
Missing	10	1.7	3	1.5	7	1.7	
Chronic illness							< 0.001
No	215	35.6	51	25.1	164	40.9	
Yes	364	60.3	143	70.4	221	55.1	
Missing	25	4.1	9	4.4	16	4.0	
Communication about financial problems with GP							0.044
No	416	68.9	135	66.5	281	70.1	
Yes	127	21.0	53	26.1	74	18.5	
Missing	61	10.1	15	7.4	46	11.5	

^a^Logistic regression analysis included individuals with complete covariate and outcome data only (*n* = 521)

^b^CRN: Any cost-related medication nonadherence in the last 12 months

^c^Differences between groups examined by chi-squared test or Fisher’s exact test^d^

Table 2 presents the results of multiple regression analysis that included individuals with complete covariate and outcome data only (*n* = 521). The chronically ill had significantly greater odds of cost-related medication nonadherence (aOR 1.96; 95% CI 1.27–3.03) than those who did not report a chronic illness. Over-indebted individuals who had discussed financial problems with their general practitioner were significantly more likely to report CRN (aOR 1.58; 95% CI 1.01–2.47) than those who never had such conversations. However, there was no significant association between CRN and other sociodemographic factors or measures of socioeconomic position in the over-indebted respondents.

**Table 2:** Crude (OR), adjusted odds ratios (aOR) and 95% confidence intervals (CI) of CRN^a^ (*n* = 521)

Variables	OR	95% CI	aOR	95% CI
Age				
18–29 years	Reference	–	Reference	–
30–49 years	0.82	0.50–1.35	0.77	0.44–1.35
50–64 years	0.67	0.38–1.17	0.58	0.30–1.12
65–79 years	0.49	0.21–1.17	0.43	0.16–1.13
Gender				
Female	Reference	–	Reference	–
Male	0.72	0.49–1.05	0.86	0.57–1.28
Migrant background				
No	Reference	–	Reference	–
Yes	1.08	0.74–1.59	1.08	0.72–1.61
Marital status				
Never married	Reference	–	Reference	–
Married	0.55	0.32–0.93	0.57	0.32–1.04
Previously married	1.00	0.67–1.51	1.04	0.61–1.76
Number of children				
No children	Reference	–	Reference	–
1 child	1.12	0.67–1.86	1.16	0.67–2.02
2 children	1.12	0.68–1.83	1.29	0.72–2.30
3 or more children	0.94	0.55–1.59	1.06	0.56–2.00
Education level				
Low	1.30	0.89–1.90	1.18	0.78–1.77
Medium	Reference	–	Reference	–
High	0.70	0.27–1.81	0.78	0.29–2.07
Employment status				
Employed	Reference	–	Reference	–
Unemployed	1.09	0.76–1.58	0.96	0.64–1.44
Chronic illness				
No	Reference	–	Reference	–
Yes	1.89	1.26–2.83	1.96	1.27–3.03
Communication about financial problems with GP				
No	Reference	–	Reference	–
Yes	1.75	1.14–2.67	1.58	1.01–2.47

^a^CRN: Any cost-related medication nonadherence in the last 12 months

## Discussion

The present study is the first to provide detailed estimates of cost-related medication nonadherence in a population of over-indebted individuals enrolled in statutory health insurance in Germany. A third of the over-indebted reported not filling prescriptions and/or skipping or decreasing doses of a prescribed medication due to financial pressures in the last year. The findings suggest that individuals’ responses to medication cost problems vary significantly by health status and patient-physician communication about financial problems. However, there was no association between cost-related medication nonadherence and other demographic characteristics or socioeconomic factors.

Thus, the results may indicate that the over-indebted often trade off payment obligations against spending on necessities such as prescribed medications – although all individuals in the present study were enrolled in statutory health insurance which covers about 90% of the German population [31].

Previous research suggests considerable variations in rates of CRN across countries and in individuals’ responses to medication cost pressures. In a recent large cross-sectional study among adults aged 55 and older the estimated prevalence of CRN in the previous year ranged from less than 4 % in France, Norway, Sweden, Switzerland, UK and Germany up to 17% in the US [12]. Individuals’ susceptibility to cost-related medication nonadherence has been found to vary by factors such as sociodemographic characteristics, and health insurance [13, 32]. More specifically, studies consistently showed that lower income reflects a primary risk factor of CRN [12, 33]. Besides income, most studies on CRN have taken a limited set of other socioeconomic indicators into account [13, 29, 33]. However, it is essential to consider that individuals at all socioeconomic positions including those with a high income may face over-indebtedness [3].

The high prevalence of CRN observed in the present study is consistent with prior estimates in various samples of individuals considered over-indebted: The prevalence of CRN ranged from 32% in a nationally representative sample of older US mortgage-delinquent adults [18] up to 65% in a cross-sectional survey of German citizens who sought debt advice agencies [19].

Few studies have explicitly examined the association between measures of over-indebtedness, including mortgage delinquency [18] and inability to pay or problems paying for medical bills [17], and CRN. This association remained significant even after control of sociodemographic characteristics, SES measures such as income and employment status, health status and health insurance. In a nationally representative sample of older US adults over-indebtedness was associated with taking less medication than was prescribed because of cost at any time in the past 2 years (aOR 8.66; 95% CI 3.72–20.16) [18]. In a sample of Arizona households, over-indebtedness was associated with cost-related delays in obtaining or inability to obtain prescribed medications (aOR 6.16; 95%-CI 3.87–9.81) [17]. Another population-based study among US adults found no association between overall indebtedness and CRN [16]. However, when types of debt were examined separately, medical debt (aOR 3.84; 95%-CI 1.70–1.12) and credit card debt (aOR 2.41; 95%-CI 1.03–5.65), as opposed to housing, automobile or student loan, were significantly associated with skipping or cutting medications due to cost even after adjustment [16].

Previous studies suggest that over-indebtedness is associated with cost-related medication nonadherence. Mechanisms that possibly explain patterns of CRN in the over-indebted population may relate to ongoing limited material resources, paralleled by psychosocial and legal stressors that those affected are assumed to face. Over-indebted individuals enrolled in statutory health insurance in Germany may not benefit from the income-related ceiling on co-payments. Those who reported delaying, not filling a prescription, skipping or decreasing doses of prescribed medications for financial reasons might have been unable to cover the amount of co-payments up to the ceiling. Others might refrain from applying for reimbursement or waiver of co-payments even when exceeding the ceiling, for reasons such as administrative barriers or disease burden. The ceiling on co-payments can be reduced to 1 % for patients with a chronic condition according to the German Social Code Book V (§62). Nevertheless, the over-indebted with a chronic condition were more likely to experience cost-related medication nonadherence than those without a chronic condition. Specifically over-indebted individuals who are employed (*n* = 307; 50.8%) might not exceed the ceiling as it is based on the gross annual household income rather than income available for living expenses including co-payments. Thus, even when income is largely spent on debt repayments to satisfy creditors, the amount of co-payments will correspond to the amount of individuals’ income only.

Therefore, CRN among the over-indebted is assumed to arise from difficulty paying for medications in the present study. On the one hand, the high prevalence of cost-related medication nonadherence may be attributable to morbidity [6–9] and associated need for medication among the over-indebted. On the other hand, medication underuse, in turn, may contribute to maintaining existing health problems. Moreover, experiences of psychological distress, social stigma and feelings of shame related to over-indebtedness [4, 5, 7] might contribute to patients’ medication underuse as a strategy to cope with medication cost. In Germany, co-payments not only apply to prescription medication but also hospital care, medical aids, rehabilitation and home nursing care unless patients actively apply for reimbursement or a waiver of co-payments that exceed the income-related ceiling. Thus, over-indebted individuals might be at increased risk of forgone medical care that goes beyond medication nonadherence [34, 35].”

Patient characteristics, medication, provider and health system factors have been suggested to influence the relationship between cost pressures and medication nonadherence [29]. In line with previous research [13, 19, 33], the present study indicates that some patient groups are at increased risk of underusing prescribed medication due to cost although all respondents were over-indebted and enrolled in statutory health insurance:

First, the chronically ill are likely to face disproportionate financial burden of medication cost, hence are more susceptible to CRN than others are. Second, studies found that patients and physicians rarely discuss medication cost problems although alternative strategies of reducing medication costs might exist in clinical practice [36–38]. In the present study, patient-physician communication about financial problems did not reflect a protective factor of CRN but was associated with greater odds of CRN. Previous findings of the role of patients’ relationship to physicians in CRN were inconsistent [36, 39].

### Limitations

A strength of this study is the high response rate of 50.2% that was achieved by recruiting participants via debt advice agencies in North Rhine-Westphalia, Germany. However, this study has some limitations. Population groups such as those who lack sufficient language skills to participate in the survey might be underrepresented. It can be assumed that those clients who experienced major difficulties paying for medications might have been either more likely to participate in the study to share their debt-related burden or decline participation. Hence, the prevalence of cost-related medication nonadherence can be both overestimated and underestimated but this potential selection bias is unlikely to affect the results of multiple logistic regression analysis. However, we need to consider that the generalizability of findings is possibly limited. Since the data was cross-sectional, further research is necessary to examine causal mechanisms in more detail. Complete case analysis might lead to imprecise results of logistic regression analysis. However, the loss of information is limited due to the small proportion of missing data, and missing values considered missing at random. Therefore, this method used to handle missing data is assumed to introduce only a minor bias. Another limitation of the study is that data on income that is a relevant risk factor of CRN was not assessed due to the difficulty to examine discretionary income when it is committed to debt payments, distrained or yet unclear to over-indebted clients. However, other standard SES measures (education, employment status) were taken into account. The annual report on over-indebtedness in Germany suggested that most of the over-indebted are not lowest-income earners but members of the middle class [3]. There are differences in administrative and legal procedures to over-indebtedness across countries [2]. Likewise, pharmaceutical policies and physicians’ means to support patients in following prescribed treatment despite cost concerns differ considerably between and within countries. Therefore, prevalence rates of CRN may vary accordingly [40–42].

Despite these limitations, the present study provides a valuable insight into patterns of CRN in the population of over-indebted individuals in Germany. This population group may face considerable barriers to access to prescribed medications even when enrolled in statutory health insurance. There is considerable evidence on adverse health effects of cost-related medication nonadherence, including increased rates of hospitalization [43] and decline in health status [21]. Given the vast number of over-indebted citizens in high-income countries, the findings suggest that cost-related medication underuse reflects a major public health problem in health systems that charge direct patient payments.

## Conclusions

Cost sharing can contribute to medication underuse due to cost, specifically in vulnerable population groups such as the over-indebted. In light of possible detrimental effects of nonadherence to prescribed medications on health, the findings of this study have important practical implications for health care, research and social policy. First, this study indicates that co-payments are often a barrier to access to medication for over-indebted individuals. Second, there is a need to monitor and evaluate the impact of cost sharing policy to prevent unmet health needs and adverse health outcomes, specifically among vulnerable population groups including over-indebted individuals. Third, raising awareness among both clinicians and debt advisors for medication underuse due to competing financial demands – including debts – may facilitate access to affordable medical care for all.

## Data Availability

The dataset generated and analysed during the current study is not publicly available due to confidentiality concerns but are available from the corresponding author on reasonable request.

## Abbreviations

aOR: adjusted Odds Ratio
CI: Confidence interval
CRN: Cost-related medication nonadherence
ICD-10-GM: German adaptation of the International Statistical Classification of Diseases and Related Health Problems
ISCED: International Standard Classification of Education
OID: Over-indebtedness survey
SES: Socioeconomic status
SHI: Statutory health insurance
